# Quantifying the Impact of Gestational Diabetes Mellitus, Maternal Weight and Race on Birthweight via Quantile Regression

**DOI:** 10.1371/journal.pone.0065017

**Published:** 2013-06-10

**Authors:** Caitlyn N. Ellerbe, Mulugeta Gebregziabher, Jeffrey E. Korte, Jill Mauldin, Kelly J. Hunt

**Affiliations:** 1 Department of Public Health Sciences, Medical University of South Carolina, Charleston, South Carolina, United States of America; 2 Center for Health Disparities Research, Medical University of South Carolina, Charleston, South Carolina, United States of America; 3 Department of Obstetrics and Gynecology, Medical University of South Carolina, Charleston, South Carolina, United States of America; University of Cincinnati, United States of America

## Abstract

**Background:**

Quantile regression, a robust semi-parametric approach, was used to examine the impact of gestational diabetes mellitus (GDM) across birthweight quantiles with a focus on maternal prepregnancy body mass index (BMI) and gestational weight gain (GWG).

**Methods:**

Using linked birth certificate, inpatient hospital and prenatal claims data we examined live singleton births to non-Hispanic white (NHW, 135,119) and non-Hispanic black (NHB, 76,675) women in South Carolina who delivered 28–44 weeks gestation in 2004–2008.

**Results:**

At a maternal BMI of 30 kg/m^2^ at the 90^th^ quantile of birthweight, exposure to GDM was associated with birthweights 84 grams (95% CI 57, 112) higher in NHW and 132 grams (95% CI: 104, 161) higher in NHB. Results at the 50^th^ quantile were 34 grams (95% CI: 17, 51) and 78 grams (95% CI: 56, 100), respectively. At a maternal GWG of 13.5 kg at the 90^th^ quantile of birthweight, exposure to GDM was associated with birthweights 83 grams (95% CI: 57, 109) higher in NHW and 135 grams (95% CI: 103, 167) higher in NHB. Results at the 50^th^ quantile were 55 grams (95% CI: 40, 71) and 69 grams (95% CI: 46, 92), respectively.

**Summary:**

Our findings indicate that GDM, maternal prepregnancy BMI and GWG increase birthweight more in NHW and NHB infants who are already at the greatest risk of macrosomia or being large for gestational age (LGA), that is those at the 90^th^ rather than the median of the birthweight distribution.

## Introduction

Obesity prior to pregnancy and high gestational weight gain (GWG) predispose women to gestational diabetes mellitus (GDM) and early onset type 2 diabetes[1]–[4]. Maternal diabetes during gestation exposes the fetus to hyperglycemia, resulting in increased fetal insulin levels that both promote the storage of excess energy as fat and act as a growth factor. Maternal diabetes has been associated with high birthweight, increased childhood and adult obesity and increased risk of type 2 diabetes[5]–[10]; however, a recent systematic review indicates that the relationship between maternal diabetes and childhood obesity is inconsistent [11]. Children exposed *in utero* to diabetes are at higher risk of obesity and diabetes than their unexposed siblings, suggesting that the increased risk to the exposed offspring is not exclusively genetic [12], [13].

Maternal obesity, high GWG and diabetes during pregnancy are modifiable risk factors that determine birthweight for gestational age. Race is an additional risk factor, albeit non-modifiable, that is associated with both diabetes prevalence and birthweight. In a previous analysis of South Carolina births 2004 through 2008 using standard regression methodology we reported a differential impact of GDM on racial differences in birthweight [14]. However, a limitation of this study was that standard regression methodology assumed that the effect of covariates was the same at all quantiles of the outcome. If, as hypothesized, the racial differences due to GDM have a greater impact in neonates already predisposed to be LGA, the estimates from standard regression models will underestimate this effect. We reported that at a delivery BMI of 35 kg/m^2^, GDM exposure was associated with an average birthweight only 17 grams (95% CI: 4, 30) higher in non-Hispanic whites (NHW), but 78 grams (95% CI: 61, 95) higher in non-Hispanic-Blacks (NHB) (controlling for gestational age, maternal age, infant sex, and having information on prenatal care). An important aspect of this finding is that our estimate of the differential impact of GDM on birthweight [60 gram racial difference (95% CI: 39, 82)] is difficult to interpret and may be underestimated because of racial differences in the distribution of birthweight.

Reference curves for fetal growth have historically been based on normality assumptions but often rely on numerous transformations and data cleaning steps [15], [16] or alternatively have been broken down into percentiles of interest [17]. Research into the association between maternal factors and birthweight has mirrored these trends. Studies either assume that normality assumptions are met and analyze the effect of maternal weight gain and diabetes on the mean birthweight, or they choose to look at these effects on infants that are considered large or small for gestational age (LGA, SGA respectively). We used both of these approaches in our recent publication [14]. In assuming normality and using standard linear regression, investigators are restricted to how factors shift the mean birthweight and are blind to whether a factor more strongly impacts infants at the lower (SGA) or upper (LGA) tail of the birthweight [18]. Alternatively, logistic regression on a SGA/LGA birthweight is often used [19] for the ease of clinical interpretation; however by dichotomizing the outcome of interest (e.g. LGA) the impact on the full distribution is ignored and results are not intuitive for individuals falling near to but at opposite sides of the cutoff point. More seriously, when the outcome is dichotomized, information is lost which can result in a loss of power, sensitivity to cutpoints, and inability to detect non-linear effects of risk factors [20]. Given that NHW and NHB infants are known to have differing birthweight distributions [17], [21], if a racial disparity in the differential impact of predictors at different quantiles of birthweight [22], [23] is hypothesized both linear and logistic regression will fail to identify this. In contrast, Quantile regression is a robust semi-parametric approach that allows birthweight to be treated continuously as in linear regression, but allows differential interpretation for the effect at different tails of the birthweight distribution as in logistic regression but with the benefit that cutoffs for LGA are empirically selected [24].

To address limitations of earlier work including our own [14], our objective was to determine the impact of GDM, obesity and GWG during pregnancy on the upper quantiles of birthweight in NHW and NHB women at the population level in South Carolina in 2004 through 2008.

## Materials and Methods

### Study Design and Population

**Figure 1:**
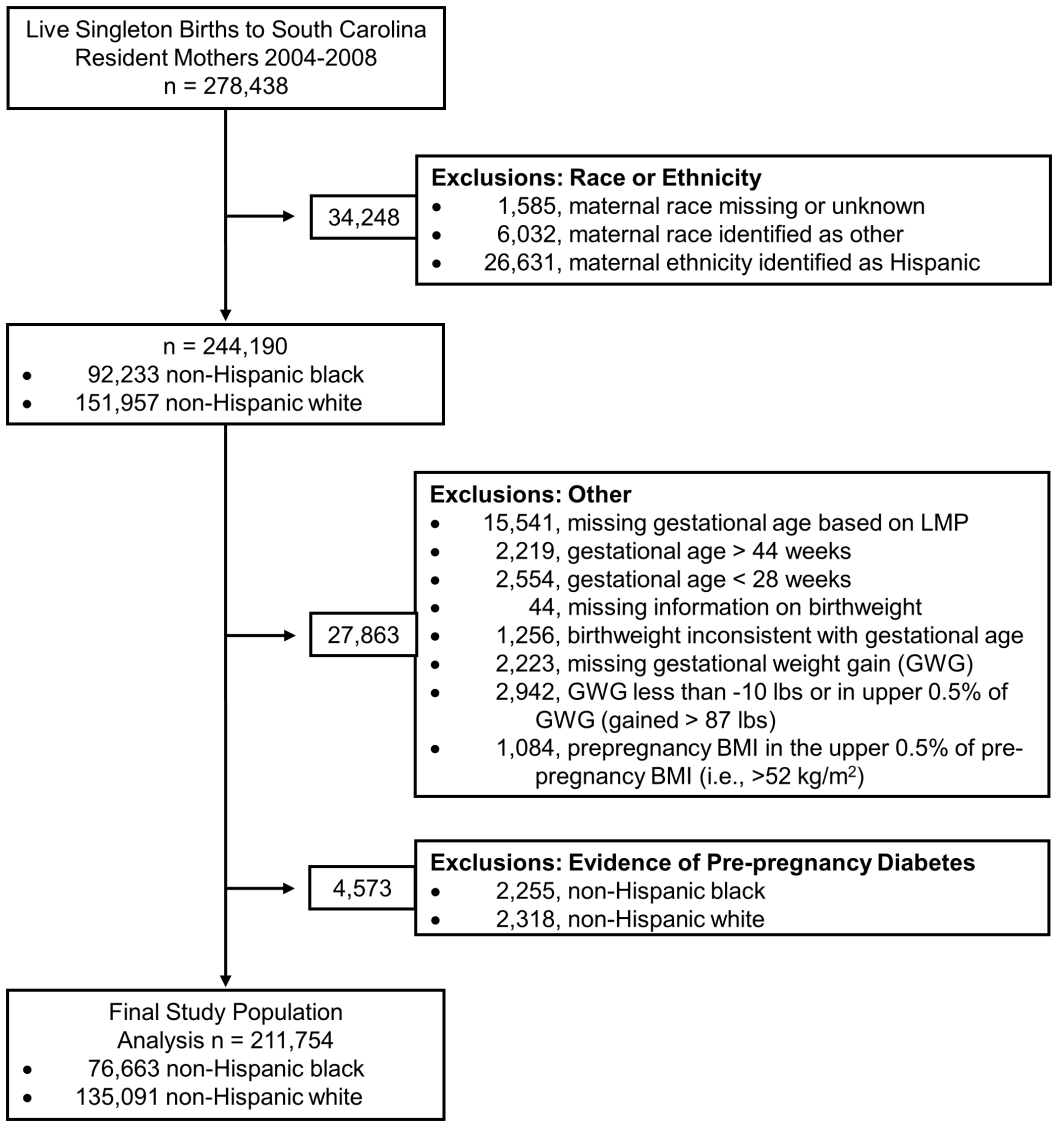
Flow chart defining study population and exclusions.

Live singleton births of South Carolina resident mothers, without prepregnancy diabetes, who self-reported their race as NHW or NHB and delivered at a gestational age of 28 to 44 weeks between January 2004 and December 2008 comprise the study population for this population-based cohort study. Selection criteria are outlined in Figure 1. Birth certificate information was obtained from the South Carolina (SC) Department of Health and Environmental Control and linked by the SC Office of Research and Statistics (ORS) to inpatient hospital discharge records for the state of SC to obtain maternal and infant inpatient procedure and diagnostic codes pertaining to delivery. Additionally, outpatient diagnostic codes were available for the prenatal period for mothers who received prenatal care through either Medicaid or the SC State Employee Health Plan. The linkage between databases is based on an algorithm developed by SC ORS and relies on personal identifying information. Maternal inpatient hospital procedure and diagnostic codes from delivery were successfully linked for 198,749 (93.86%) births, while prenatal information was available for 110,926 (52.38%) births to mothers with Medicaid and 13,785 (6.51%) births to mothers with the State Employee Health Plan.

The Institutional Review Board of the Medical University of South Carolina approved the study as exempt research (HR Number 19410, August 25, 2009 to August 25, 2014) and waived the need for informed consent given the use of routinely collected de-identified patient data for this analysis.

### Data Collection

Information was obtained from the birth certificate, inpatient hospital discharge records pertaining to delivery (i.e., maternal diagnostic codes) and prenatal care records available for those women who received prenatal care through either Medicaid or the SC State Employee Health Plan.

Diabetes during pregnancy was defined by GDM or pre-pregnancy diabetes reported on the birth certificate, or coded for on medical records including the inpatient hospital discharge records or prenatal care. The prenatal period was defined by the date of delivery and gestational age of the infant at delivery and additionally included the year prior to conception in defining pre-pregnancy diabetes. Additionally, for a diagnosis of diabetes during pregnancy based on the prenatal data alone, two or more ICD-9-CM diagnostic codes indicative of diabetes were required in the medical record. This criterion was based on a validated algorithm developed for use in the Veterans Health Administration [25]. Primary and secondary inpatient hospital and prenatal ICD-9-CM diagnosis codes used to define diabetes included those for pre-pregnancy and GDM (i.e., 64801–64802, 25000–25092, 64881–64882). Further classification into having pre-pregnancy or GDM was based on evidence of pre-pregnancy diabetes from any source. Hence, when one source reported pre-pregnancy diabetes and another reported GDM, a woman was classified as having pre-pregnancy diabetes. All women classified as having pre-pregnancy diabetes were excluded from the current analysis.

Maternal BMI was calculated as mother’s pre-pregnancy weight in kilograms divided by height in meters squared. GWG was calculated as weight at delivery (which included the weight of the infant and products of conception) minus mother’s pre-pregnancy weight. Maternal hypertension during pregnancy was defined as reported on the birth certificate (i.e., either gestational or pre-pregnancy). Information to calculate maternal pre-pregnancy BMI and GWG was obtained from the birth certificate.

Birthweight, maternal race, education (dichotomized based on high school graduation or GED), private health insurance status, first born (based on report of previous live births) and tobacco use were defined as reported on the birth certificate. Adequacy of prenatal care was dichotomized based on the revised GINDEX and further categorized women into those with adequate (i.e., intensive, adequate or intermediate utilization) or inadequate (i.e., inadequate utilization or no care) care. The GINDEX combines information from the birth certificate on the trimester when prenatal care was first received and the total number of prenatal visits [26]. Birthweights inconsistent with gestational age were identified based on a modified version of the criteria published by Alexander et al. [27] with the modification allowing for birthweight up to 6500 grams at 39 weeks and 7000 grams at 40 or more weeks. We conducted analyses of birthweight adjusted for gestational age, representing a measure of fetal growth on a continuous scale. We defined gestational age based on reported date of last menstrual period (LMP) as reported on the birth certificate.

### Statistical Analysis

Quantile regression [28] was used to investigate the impact of race, diabetes status, GWG and maternal BMI on an infant’s birthweight controlling for gestational age (i.e., a continuous measure of fetal growth). This method was preferred over ordinary least squares regression because it allows for the independent factors of interest and any confounders to have a different effect magnitude or direction at different parts of the birthweight distribution (50%, 75% and 90%, etc.) In addition this approach is robust in that it makes no distributional assumptions about the dependent variable (i.e., infant birthweight). Finally, when the normality assumption is met, results from quantile regression for the 50^th^ quantile are equivalent to results obtained for standard linear regression (i.e. median is equivalent to mean).

To model this relationship we examined the covariates of interest using univariate analysis and final models were decided upon based on known confounders in the literature and model building exercises using Aikike’s Information Criteria (AIC) as a goodness of fit measure. Functional forms of covariates (i.e. 1^st^ vs. 2^nd^ order polynomial) were considered as part of model building. Due to the complexity of the relationship of interest two final models were considered.

The first model (referred to as Model 1) investigates the interaction of maternal race, GDM status, and maternal prepregnancy BMI (modeled as a second-order polynomial) and their association with infant birthweight, controlling for infant sex, maternal age, weeks gestation, adequacy of prenatal care, tobacco use, hypertension diagnosis, first born status, indicator for available data on prenatal care, and GWG modeled as a third-order polynomial. The second model (referred to as Model 2) investigates the interaction of maternal race, GDM status, and GWG (modeled as a third-order polynomial) and their association with infant birthweight, controlling for infant sex, maternal age, weeks gestation, adequacy of prenatal care, tobacco use, hypertension diagnosis, first born status, indicator for available data on prenatal care, and maternal prepregnancy BMI modeled as a second-order polynomial. Interactions were specified *a priori* based on previous literature and the aims of the study. Analyses were performed using R version 2.10.1 and the package quantreg [29]. Reported quantiles were chosen based on clinical significance.

Despite the fact that we were interested in GWG and fetal growth which are both directly related to gestational age, we included live births throughout the third trimester (i.e., gestational age of 28 to 44 weeks) rather than limiting our population to term pregnancies to increase the generalizability of our results. As a result, throughout our analyses we adjusted for gestational age and examined GWG as a continuous variable.

## Results

### Population Characteristics

The prevalence of GDM was similar in NHB (6.2%) and NHW (6.3%) women (Table 1). NHW mothers were on average older than NHB mothers, and mothers with GDM were older than mothers without diabetes. In addition, NHB mothers had a higher pre-pregnancy BMI on average than NHW mothers and mothers with GDM had a higher pre-pregnancy BMI than mothers without diabetes. However, this trend was not observed for GWG, with NHW mothers without diabetes gaining slightly more than the other three groups.

**Table 1:** Characteristics of the study population stratified by race and diabetes status for singleton live births of 28 through 44 weeks gestational age in South Carolina 2004–2008.

	Non-Hispanic White		Non-Hispanic Black	
	No DM	GDM	No DM	GDM
	N = 126,524	N = 8,567	N = 71,939	N = 4,724
Infant Characteristics				
Birthweight(g)	3340(525)	3386(567)	3072(532)	3184(602)
Male infant (%)	51.20	53.11	50.64	50.72
Gestational age (weeks)	38.7(2.0)	38.4(2.0)	38.3(2.3)	38.0(2.3)
Maternal Characteristics				
Maternal Age (years)	27.0(5.9)	29.6(5.9)	24.3(5.6)	27.5(6.2)
BMI (kg/m^2^)	25.7(6.1)	29.3(7.3)	28.1(7.0)	31.7(7.5)
Gestational weight gain (kg)	13.7(7.6)	11.7(7.7)	11.1(8.0)	11.5(8.3)
Inadequate Prenatal Care (%)	9.15	6.96	14.48	8.58
Tobacco Use (%)	18.14	16.70	8.17	7.95
Hypertension (%)	6.36	14.25	7.59	17.58
First born (%)	44.30	38.25	40.49	34.50
Prenatal Medical Records (%)	48.29	46.67	75.35	76.31

Statistics reported are means (sd) or percentages.

### Quantile Regression

#### Quantile regression: GDM overview of model fitting

Using quantile regression, infant sex, age, weeks gestation, adequate prenatal care, tobacco use, hypertension status, first born status, and having prenatal medical records available were significant predictors at the 50^th^, 75^th^ and 90^th^ quantile with the exception of adequate prenatal care at the 90^th^ quantile in both Model 1 and Model 2. In addition, all of the singular effects of GDM, race, GWG and prepregnancy BMI as well as some of the two and three way interactions for maternal BMI and GWG were significant at the 50^th^, 75^th^ and 90^th^ quantiles in both models. (See table S1 for model details).

#### Quantile regression: GDM and maternal prepregnancy BMI as predictors of birthweight

**Figure 2:**
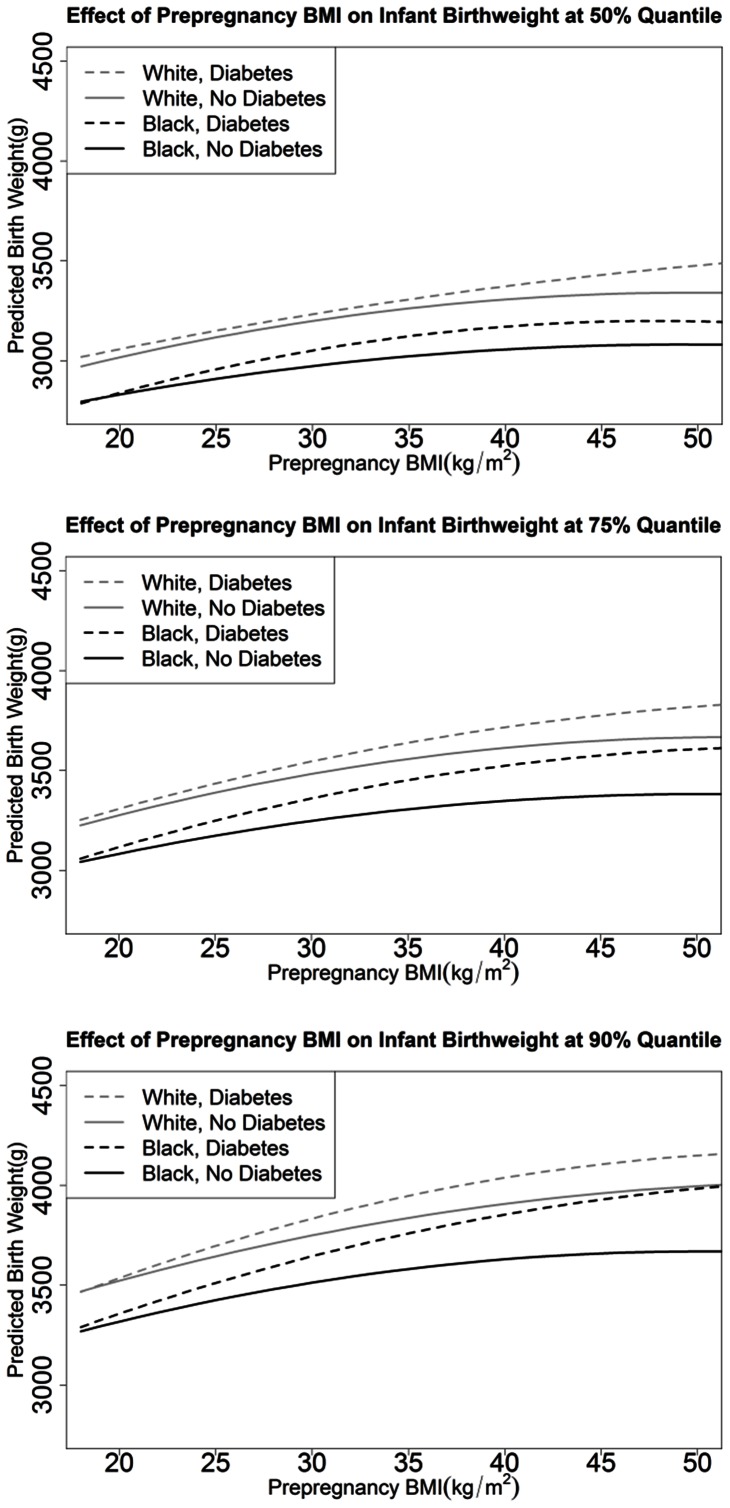
Predicted Infant Birthweight using model 1. Results pictured for mother’s age 26, gestational age 38 weeks, 11.3 kg (25 lbs) weight gain, averaged over effects of infant sex, prenatal care, smoking, hypertension, first born, and availability of prenatal information at the 50^th^ (A), 75^th^ (B), and 90^th^ (C) quantile.

Figure 2 displays the predicted birthweight at the 50^th^, 75^th^, and 90^th^ quantile given the mother’s prepregnancy BMI (Model 1) and assuming a mother’s age of 26, gestational age of 38 weeks, GWG of 11.3 kg (25 lbs), and averaged over dichotomous factors. NHW mothers gave birth to heavier babies than NHB mothers. Mothers with GDM had heavier babies than mothers without diabetes with the difference generally increasing with increasing quantile of birthweight and with increasing maternal BMI. In addition, the impact of GDM on birthweight (i.e., the difference in infant birthweight between a mother with GDM and a mother without diabetes) was greater in NHB mothers than NHW mothers. At the upper tail of the predicted birthweight distribution (90^th^ quantile) as compared to the median, the impact of GDM on birthweight was greater (i.e., the difference in infant birthweight between a mother with GDM and a mother without diabetes) and the impact of prepregnancy BMI on birthweight was greater (i.e., slope of the curves is greater); however, racial differences in the impact of GDM on birthweight appear similar across birthweight quantiles (except possibly when maternal prepregnancy BMI is very high). At a maternal BMI of 30 kg/m^2^ at the 50^th^ quantile of birthweight, exposure to GDM *in utero* was associated on average with birthweights 34 grams (95% CI: 17, 51) higher in NHW and 78 grams (95% CI: 56, 100) higher in NHB (Table 2). Parallel numbers at the 90^th^ quantile were 84 grams (95% CI: 57, 112) in NHW and 132 grams (95% CI: 104, 161) in NHB. Hence, GDM had a greater impact on birthweight in NHB infants and NHW infants at the 90^th^ than 50^th^ quantile [50 gram difference in NHW (i.e., 84–34) and 54 gram difference in NHB (i.e., 132–78)].

**Table 2:** Model One: Predicted infant birth weights (gm) and birth weight differences (with 95% CI) at a maternal pre-pregnancy body mass index of 25, 30, 35, and 45 kg/m^2^ in NHW and NHB with and without diabetes.

PrepregnancyBMI (kg/m^2^)	Quantile	non-Hispanic white			non-Hispanic black			Racial Difference
		No Diabetes	GDM	±GDM	No Diabetes	GDM	±GDM	
25	50%	3117	3149	33	2909	2957	48	15
		(3110,3124)	(3135,3164)	(19,47)	(2901,2917)	(2934,2980)	(25,70)	(−12,41)
	75%	3389	3434	46	3173	3248	75	29
		(3381,3397)	(3418,3450)	(31,61)	(3164,3182)	(3225,3271)	(53,97)	(2,56)
	90%	3644	3696	52	3424	3510	86	34
		(3633,3655)	(3670,3722)	(28,77)	(3412,3436)	(3486,3535)	(63,110)	(0,68)
30	50%	3198	3233	34	2973	3051	78	44
		(3191,3206)	(3215,3250)	(17,51)	(2964,2981)	(3029,3073)	(56,100)	(16,72)
	75%	3483	3545	62	3248	3360	112	50
		(3474,3491)	(3525,3565)	(42,82)	(3238,3257)	(3335,3385)	(88,137)	(19,82)
	90%	3749	3833	84	3512	3644	132	48
		(3737,3760)	(3805,3861)	(57,112)	(3499,3525)	(3616,3673)	(104,161)	(9,88)
35	50%	3261	3307	45	3022	3122	100	55
		(3253,3270)	(3288,3326)	(26,64)	(3013,3030)	(3098,3146)	(76,125)	(24,86)
	75%	3557	3638	81	3306	3452	146	65
		(3548,3567)	(3616,3661)	(59,103)	(3295,3316)	(3425,3479)	(119,173)	(30,100)
	90%	3836	3947	110	3580	3759	178	68
		(3824,3849)	(3916,3978)	(79,142)	(3567,3594)	(3727,3790)	(147,210)	(24,112)
45	50%	3333	3429	96	3076	3195	119	23
		(3313,3352)	(3390,3468)	(53,139)	(3062,3089)	(3151,3239)	(74,165)	(−39,85)
	75%	3649	3776	127	3373	3575	203	75
		(3631,3667)	(3733,3819)	(82,173)	(3355,3390)	(3524,3626)	(150,255)	(6,145)
	90%	3960	4105	145	3659	3928	269	124
		(3931,3989)	(4024,4185)	(60,230)	(3631,3686)	(3855,4001)	(192,346)	(10,239)

Results listed for maternal age of 26, gestational age 38 weeks, gestational weight gain 11.25 kg, averaged over effects for dichotomous factors.

#### Quantile regression: GDM and GWG as predictors of birthweight

**Figure 3:**
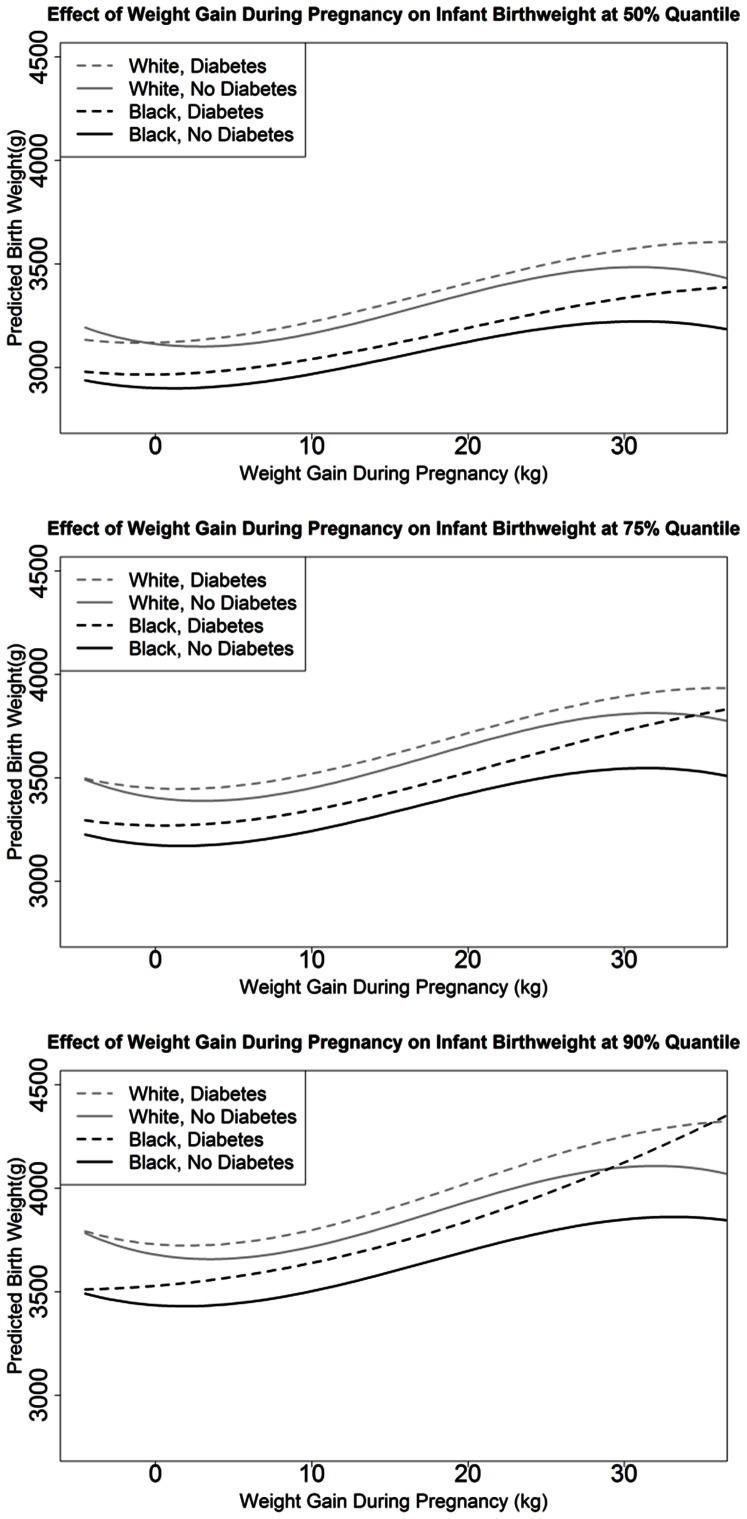
Predicted Infant Birthweight using model 2. Results pictured for mother’s age 26, gestational age 38 weeks, prepregnancy BMI 30, averaged over effects of infant sex, prenatal care, smoking, hypertension, first born, and availability of prenatal information at the 50^th^ (A), 75^th^ (B), and 90^th^ (C) quantile.

Figure 3 displays the predicted birthweight at the 50^th^, 75^th^, and 90^th^ quantile given the mother’s GWG (Model 2) and assuming a mother’s age of 26, gestational age of 38 weeks, prepregnancy BMI of 30 kg/m^2^, and averaged over dichotomous factors. Mothers with GDM had heavier babies than mothers without diabetes with the difference remaining relatively stable across most of the distribution of GWG: exceptions being at very low GWG (i.e., weight loss during pregnancy) or when GWG exceeded 20 kg. In the second model at the upper tail of the predicted birthweight distribution (90^th^ quantile) as compared to the median, the impact of GDM on birthweight was greater in NHB, but not in NHW. Similarly, at the upper tail of the predicted birthweight distribution (90^th^ quantile) as compared to the median, the impact of GWG on birthweight appeared greater in NHB with GDM than in the other three groups of women (i.e., NHW with GDM, NHW without diabetes and NHB without diabetes). As a result, in model 2 racial differences in the impact of GDM on birthweight appear at the upper tail of the predicted birthweight distribution (90^th^ quantile), but not at the median. When GWG is 13.5 kg (i.e., 30 pounds) at the 50^th^ quantile of birthweight, exposure to GDM *in utero* was associated on average with birthweights 55 grams (95% CI: 40, 71) higher in NHW and 69 grams (95% CI: 46, 92) higher in NHB (Tables 3). Parallel numbers at the 90^th^ quantile were 83 grams (95% CI: 57, 109) in NHW and 135 grams (95% CI: 103, 167) in NHB. Hence, GDM had a greater impact on birthweight in NHB infants (66 gram difference), but a similar impact in NHW infants (28 gram difference), comparing the 90^th^ to 50^th^ quantile of birthweight.

**Table 3:** Model Two: Predicted infant birth weights (gm) and birth weight differences (with 95% CI) at gestational weight gain (GWG) of 9, 13.5, 18, and 30 kg in NHW and NHB with and without diabetes.

GWG (kg)	Quantile	non-Hispanic white			non-Hispanic black			Racial Difference
		No Diabetes	GDM	±GDM	No Diabetes	GDM	±GDM	
9	50%	3149	3204	55	2954	3027	73	18
		(3141,3156)	(3186,3221)	(38,72)	(2946,2962)	(3001,3053)	(47,99)	(−13,49)
	75%	3435	3505	69	3228	3330	102	32
		(3426,3444)	(3484,3525)	(49,89)	(3219,3237)	(3299,3360)	(71,132)	(−4,69)
	90%	3702	3782	80	3487	3624	137	56
		(3690,3713)	(3756,3808)	(55,105)	(3475,3500)	(3585,3662)	(98,175)	(10,102)
13.5	50%	3226	3281	55	3019	3088	69	13
		(3219,3233)	(3265,3297)	(40,71)	(3012,3027)	(3065,3111)	(46,92)	(−14,41)
	75%	3516	3581	66	3302	3399	97	32
		(3507,3524)	(3564,3599)	(49,82)	(3293,3311)	(3372,3427)	(70,124)	(−0,63)
	90%	3785	3868	83	3565	3700	135	52
		(3774,3796)	(3841,3895)	(57,109)	(3553,3577)	(3668,3732)	(103,167)	(11,94)
18	50%	3317	3368	51	3092	3158	66	15
		(3309,3324)	(3349,3387)	(32,70)	(3083,3101)	(3131,3186)	(39,94)	(−18,48)
	75%	3613	3674	60	3387	3485	98	38
		(3605,3622)	(3655,3692)	(42,79)	(3377,3396)	(3450,3519)	(63,133)	(−2,77)
	90%	3889	3975	86	3656	3794	138	52
		(3878,3900)	(3934,4016)	(45,127)	(3643,3669)	(3757,3832)	(100,176)	(−4,107)
25	50%	3442	3498	56	3189	3269	80	24
		(3433,3450)	(3468,3528)	(26,87)	(3177,3200)	(3226,3311)	(37,124)	(−29,77)
	75%	3753	3817	64	3502	3630	127	64
		(3743,3763)	(3790,3843)	(37,90)	(3490,3515)	(3570,3689)	(67,187)	(−2,130)
	90%	4040	4149	109	3789	3974	185	76
		(4027,4054)	(4087,4210)	(47,171)	(3773,3806)	(3915,4033)	(124,245)	(−10,162)
30	50%	3484	3568	85	3221	3335	114	29
		(3472,3495)	(3535,3601)	(50,119)	(3204,3238)	(3243,3427)	(21,207)	(−70,128)
	75%	3809	3895	86	3545	3728	184	97
		(3795,3822)	(3842,3948)	(32,140)	(3526,3563)	(3596,3860)	(50,317)	(−46,241)
	90%	4102	4252	150	3850	4126	276	126
		(4084,4120)	(4183,4321)	(80,220)	(3824,3875)	(4002,4249)	(150,402)	(−18,270)

Results listed for maternal age of 26, gestational age 38 weeks, prepregnancy BMI 30, averaged over effects for dichotomous factors.

#### Quantile regression: GDM as a predictor of birthweight across all birthweight quantiles

**Figure 4:**
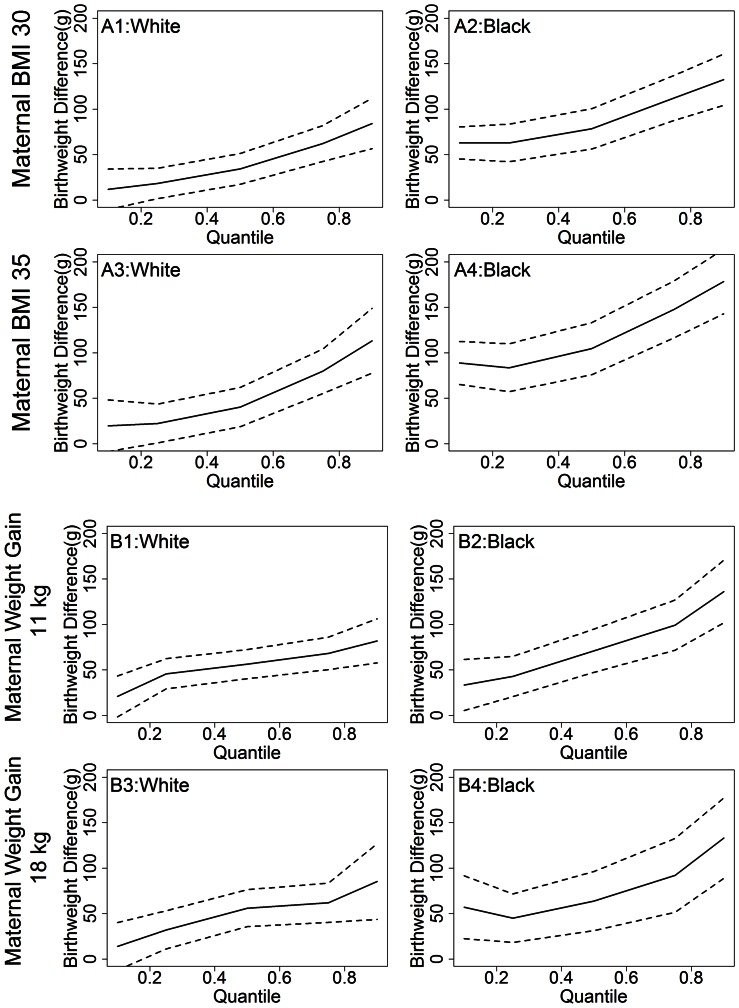
Effect of GDM on Birthweight by Quantile. Effect of GDM on birthweight in Model One (A) and Model Two (B). The figure presents the additional birthweight associated with a mother having GDM in NHW and NHB for a maternal BMI of 30 or 35 (A) or gestational weight gain of 13.5 or 18 kg (B).

Figure 4 presents the effect of GDM on infant birthweight in NHB and NHW mothers over the entire distribution of predicted birthweight. In this figure we can see that in both models GDM has a greater contribution to birthweight in large babies (i.e., upper quantiles of birthweight) as compared with small babies (i.e., lower quantiles of birthweight). Additionally, analyses that rely on standard regression would underestimate the effects of GDM and race on birthweight for LGA infants. The racial disparity in the impact of GDM on infant birthweight, with GDM having a greater impact in NHB than NHW, was evident when modeled as a three-way interaction with maternal pre-pregnancy BMI (Model 1). Analyses using standard regression would in addition underestimate the rate of increased racial disparity as quantile of birthweight increased (i.e. the relative difference in slopes for figure A1 vs. A2 in figure 4). In contrast, when modeled as a three-way interaction with GWG (Model 2), point estimates for the effect of GDM on infant birthweight appear higher in NHB than NHW only at the upper end of the birthweight distribution, but similar across the remainder of the distribution.

## Discussion

Mothers with GDM have heavier babies than mothers without diabetes[30]–[33] and work including our own indicates that the impact of GDM may be greater in NHB than in NHW [14], [34]. Previous studies have also demonstrated that infant birthweight is affected by maternal prepregnancy BMI and GWG. This study extends the current literature, by using quantile regression to examine the impact of GDM on birthweight across birthweight quantiles with a focus first on maternal prepregnancy BMI and second on GWG. We report that mothers with GDM had heavier babies than mothers without diabetes with the difference generally increasing with increasing birthweight quantile and with increasing maternal BMI. Moreover, across the continuum of maternal BMI we observed racial differences in the impact of GDM on birthweight which appeared similar across birthweight quantiles (except possibly when maternal prepregnancy BMI was very high). Focusing on GWG we report that the impact of GDM on birthweight is greater in NHB than NHW at the upper tail of the birthweight distribution. Moreover, at the upper tail of the birthweight distribution the impact of GWG on birthweight is greatest in NHB with GDM. Hence, GDM, maternal prepregnancy BMI and GWG have a greater impact on birthweight at the upper tail of the birthweight distribution (i.e., 90^th^ quantile) as compared to the median. Moreover, GDM and GWG have the greatest impact on racial difference in birthweight at the upper tail of the birthweight distribution (i.e., 90^th^ quantile).

Our results are consistent with and build off our recent article as well as results from the Hyperglycemia and Adverse Pregnancy Outcomes Study (HAPO) which reported hyperglycemia below levels diagnostic of GDM [35], and maternal BMI at the time of the oral glucose tolerance test (OGTT) [36], [37] were each independently and positively associated with birthweight greater than the 90^th^ percentile. Interestingly, our study provides evidence that the impact of GDM and maternal pre-pregnancy BMI is greater at the upper tail of the birthweight distribution; hence, GDM, prepregnancy BMI and GWG have a greater impact on birthweight in babies who are already the heaviest.

One limitation of our study is the use of administrative databases and the reliability of data obtained from these databases. In 2004 the South Carolina birth certificate was revised: check boxes were added to differentiate between gestational and established diabetes; and information on maternal height, pre-pregnancy weight and weight at delivery was added. Moreover, a validation study conducted on a population-based sample of 4,541 women in Washington State (which uses a comparable birth certificate) compared information combined across birth certificate and hospital discharge data to medical record review, reporting a true positive fraction of 93.3 (95% CI, 86.9, 99.7) and a false positive fraction of 0.9 (95% CI: 0.5, 1.4) [38] for GDM. Previous studies have validated the reliability of maternal BMI from birth certificates [39]–[41], with high correlation between self-report and clinically measured pre-pregnancy BMI that do not seem to differ by race/ethnicity, gestational age, or weight itself [42]. Nevertheless, the possible limitations in the quality of our birth certificate data led us to exclude women with BMI<15 kg/m^2^ in the current analysis. With regard to GWG, a few studies have examined data reliability with encouraging results: high concordance was found between self-reported and clinically recorded weight [41] as well as between birth certificate data and clinically recorded GWG [43]. Additionally, because timing and consistency of prenatal care may be associated with the quality of data pertaining to maternal BMI, GWG and gestational age, we have controlled for prenatal care throughout all analyses using the revised GINDEX [26]. We therefore believe misclassification of maternal BMI and GWG to be minimal. Finally, the use of LMP to calculate gestational age has limitations [44] which may be differential with respect to diabetes or obesity status given the association between diabetes, obesity and irregular menses [45], [46]; however, because birthweight, our outcome, may differentially impact the clinical/obstetric estimate of gestational age we relied on LMP to calculate gestational age.

Another limitation of our study is that we did not have information on treatment received for diabetes during pregnancy or maternal compliance to offered treatments– an important factor because tight control of glucose levels during pregnancy impacts infant birthweight [47], [48]. In fact, our finding of a greater impact of GDM on birthweight at the 90^th^ quantile as compared to the median may be a result of higher maternal compliance to GDM treatments among women with infants of median birthweight. Moreover, the quality of data on diabetes status is impacted not only by reporting, coding and screening practices (both before and during pregnancy), but by whether or not we had information on prenatal care from either the State Employee Health Plan or Medicaid, all of which may be differential with respect to race. In our study use of Medicaid was higher in NHB (73.0%) than NHW (40.7%); hence, ascertainment of diabetes during pregnancy may be more complete in NHB than NHW. On the other hand, because Medicaid eligibility is dependent on need and eligibility increases during pregnancy, individuals with private insurance may receive better care prior to pregnancy. In this study the prevalence of GDM was similar in NHW and NHB (6.3 vs. 6.1%) women. We elected to classify and exclude individuals as having pre-pregnancy diabetes if information from any source reported pre-pregnancy diabetes. Our prevalence estimates for GDM are consistent with results from a study of the Kaiser Permanente managed health care program in Southern California which reports 2005 prevalence estimates for GDM of 5.3% in NHW and 5.0% in NHB [49].

A final limitation of the study is that, by using quantile regression, the cut-point is empirically rather than clinically derived. As a result, care must be taken when interpreting the results across several studies. In addition, in contrast to linear or logistic regression the interpretation is further complicated by having to interpret the results for each quantile of the distribution rather than report a single summary measure of effect. However, given the size of our sample and the additional insight provided by quantile regression the strengths of this analytical method outweigh the limitations.

Potential explanations for racial differences of the impact of GDM on birthweight are that a higher percentage of NHB than NHW women with GDM actually had undiagnosed diabetes prior to pregnancy, that diabetes with onset during pregnancy is actually more severe in NHB than NHW women, or that treatment (or response to treatment) for GDM is poorer in NHB than NHW women. The prevalence of intensive prenatal care as defined by the revised GINDEX was higher in NHB women (i.e., 25.5%) than NHW women (i.e., 21.7%) with GDM. However, NHW women with GDM were much more likely to report private health insurance than NHB women with GDM (i.e., 57.3% versus 28.3%) which may indicate more comprehensive health care prior to pregnancy in NHW than NHB.

Given the high prevalence of obesity and diabetes in childbearing women and the high frequency of excessive GWG [50], it is important to understand their association with tangible infant outcomes. The 2009 Institute of Medicine Guidelines for Gestational Weight Gain do not consider GDM as a potential modifying factor because the relationship between GWG, GDM and maternal/infant outcomes remains unclear [51]. Recently, in separate analyses we reported that the impact of GDM during pregnancy may be greater in NHB than in NHW [14], but that we did not find strong evidence for racial differences in the association between maternal BMI and birthweight [50], or in the association between GWG and birthweight [50]. In the current study, our findings indicate that the impact of GDM on birthweight increases with increasing maternal pre-pregnancy BMI and with increasing birthweight quantiles in NHW and NHB women. Additionally, in NHB women the impact of GDM on birthweight increases at the upper end of the GWG distribution. Finally, GDM and GWG have the greatest impact on racial difference in birthweight at the upper tail of the birthweight distribution (i.e., 90^th^ quantile).

Our findings may be clinically important if GWG can be considered as a marker of diabetes control during pregnancy. High GWG appears to have a greater impact on birthweight in women with GDM than in women without diabetes, indicating it may be a marker of poor diabetes control and potentially a factor used to identify infants at highest risk of being LGA. This difference appears greatest at the upper tail of the birthweight distribution among infants at greatest risk of macrosomia or being LGA. Moreover, GDM appears to have the greatest impact on racial differences in birthweight at the upper tail of the birthweight distribution in women with high GWG, indicating that limiting GWG (i.e., improving diabetes control) in these pregnancies may reduce racial differences of the impact of GDM on birthweight.

In summary, our findings with respect to birthweight quantile are important because they indicate that GDM, maternal prepregnancy BMI and GWG increase birthweight more in NHW and NHB infants who are already at the greatest risk of macrosomia or being LGA, that is those at the 90^th^ quantile of the birthweight distribution. In other words, the impact of these exposures on birthweight appears greatest in those at highest risk of macrosomia. Clinically, our findings indicate women with GDM and additional risk factors for having a large infant (i.e., high GWG, high pre-pregnancy BMI as well as other factors that put them at high risk) appear at particularly high risk of having a LGA infant or infant with macrosomia. The potential public health impact of these findings is high given the high prevalence of maternal GDM, obesity and excessive GWG. Further research is warranted not only to understand the impact of GDM, maternal prepregnancy obesity and GWG on infant birthweight, infant health and racial health disparities, but to develop interventions which target high risk women and their infants.

## Supporting Information

Table S1 (PDF)
