# Supplementary material for: Quantifying the Impact of Gestational Diabetes Mellitus, Maternal Weight and Race on Birthweight via Quantile Regression
Source: PLoS One. 2013 Jun 10;8(6):e65017. doi: 10.1371/journal.pone.0065017 (PMC3677894; doi:10.1371/journal.pone.0065017)
Supplement: Table S1 — (PDF) [file pone.0065017.s001.pdf]

Table S1: Regression Coefficients from Models One and Two

Quantile	***Model One: Diabetes, Race and BMI Interaction***			***Model Two: Diabetes, Race and GWG Interaction***		
	50%	***75%***	***90%***	***50%***	***75%***	***90%***
Intercept	3366.70(3.40)	3634.56(3.88)	3884.61(5.19)	3355.57(3.32)	3625.21(3.92)	3875.36(5.14)
Male Infant	121.80(2.21)	130.59(2.54)	138.98(3.42)	121.73(2.16)	131.01(2.52)	139.23(3.41)
Maternal Age (years)	2.97(0.22)	3.76(0.26)	4.60(0.34)	2.91(0.21)	3.71(0.25)	4.49(0.34)
GA (weeks)	102.43(0.61)	89.79(0.92)	75.84(0.93)	102.41(0.61)	89.68(0.91)	75.57(0.91)
GA^2($weeks^2$)	-11.86(0.16)	-8.95(0.29)	-4.90(0.24)	-11.84(0.16)	-8.95(0.28)	-4.92(0.23)
Inadequate Prenatal Care	-21.10(3.62)	-12.50(3.98)	-8.14(5.76) *	-23.25(3.50)	-14.11(4.01)	-8.99(5.58) *
Tobacco Use	-164.55(3.24)	-168.43(3.73)	-175.21(5.36)	-165.90(3.18)	-167.77(3.76)	-174.25(5.26)
Hypertension	-158.64(4.79)	-150.95(5.47)	-138.14(6.87)	-157.65(4.51)	-149.77(5.08)	-136.34(6.49)
First born	-73.92(2.45)	-68.09(2.83)	-65.84(3.79)	-75.01(2.38)	-67.25(2.78)	-65.00(3.75)
Prenatal MR	-40.44(2.53)	-34.58(2.91)	-23.30(3.87)	-39.51(2.47)	-34.97(2.88)	-22.45(3.86)
Diabetes	34.23(8.67)	62.09(10.08)	84.20(14.13)	56.41(8.17)	68.12(9.12)	81.94(12.36)
Black	-225.58(3.58)	-235.05(4.13)	-236.75(5.53)	-199.63(3.29)	-209.67(3.89)	-216.44(5.28)
GWG (kg)	16.46(0.26)	17.66(0.30)	18.47(0.39)	17.39(0.31)	18.19(0.37)	18.93(0.49)
GWG^2 (kg^2)	0.49(0.02)	0.58(0.02)	0.66(0.03)	0.57(0.03)	0.65(0.03)	0.75(0.04)
GWG^3 (kg^3)	-0.03(0.00)	-0.03(0.00)	-0.03(0.00)	-0.03(0.00)	-0.04(0.00)	-0.04(0.00)
BMI (kg/m^2)	14.49(0.29)	16.87(0.29)	19.25(0.44)	13.29(0.19)	15.78(0.23)	18.19(0.31)
BMI^2 ($(kg/m^2)^2$)	-0.37(0.03)	-0.39(0.03)	-0.35(0.05)	-0.36(0.02)	-0.38(0.02)	-0.36(0.03)
Diabetes:Race	44.02(14.24)	50.23(16.06)	48.15(20.12)	14.59(14.57)*	31.11(16.72)*	54.28(21.39)
Diabetes:BMI	1.24(0.90)*	3.53(0.87)	5.82(1.67)			
Diabetes:BMI^2	0.19(0.10)*	0.05(0.09)*	-0.12(0.20)*			
Diabetes:GWG				-0.07(1.20)*	-0.90(1.43)*	0.49(2.52)*
Diabetes:GWG^2				-0.21(0.11)*	-0.12(0.09)*	-0.06(0.19)*
Diabetes:GWG^3				0.02(0.01)	0.01(0.01)*	0.01(0.01)*
Race:BMI	-3.21(0.39)	-3.62(0.43)	-3.64(0.63)			
Race:BMI^2	0.07(0.04)*	0.06(0.05)*	-0.04(0.07)*			
Race:GWG				-2.72(0.51)	-1.50(0.59)	-1.45(0.80)*
Race:GWG^2				-0.23(0.04)	-0.21(0.05)	-0.22(0.06)
Race:GWG^3				0.01(0.00)	0.01(0.00)	0.01(0.00)
Diabetes:Race:BMI	4.01(1.67)	3.58(1.64)	3.39(2.26)*			
Diabetes:Race:BMI^2	-0.36(0.17)	-0.13(0.17)*	0.11(0.27)*			
Diabetes:Race:GWG				-1.00(2.36)*	-0.14(2.99)*	-0.96(3.87)*
Diabetes:Race:GWG^2				0.20(0.16)*	0.15(0.19)*	-0.01(0.29)*
Diabetes:Race:GWG^3				-0.01(0.01)*	0.00(0.02)*	0.01(0.02)*

*Indicates the regression coefficient was not statistically different from zero at p=0.05 level.
